# Understanding intestinal health in nursery pigs and the relevant nutritional strategies

**DOI:** 10.5713/ab.21.0010

**Published:** 2021-02-14

**Authors:** Sung Woo Kim, Marcos E. Duarte

**Affiliations:** Department of Animal Science, North Carolina State University, Raleigh, NC 27695, USA

**Keywords:** Inflammation, Intestinal Health, Microbiota, Nutritional Strategies, Nursery Pigs, Oxidative Stress

## Abstract

In the modern pig production, pigs are weaned at early age with immature intestine. Dietary and environmental factors challenge the intestine, specifically the jejunum, causing inflammation and oxidative stress followed by destruction of epithelial barrier and villus structures in the jejunum. Crypt cell proliferation increases to repair damages in the jejunum. Challenges to maintain the intestinal health have been shown to be related to changes in the profile of mucosa-associated microbiota in the jejunum of nursery pigs. All these processes can be quantified as biomarkers to determine status of intestinal health related to growth potential of nursery pigs. Nursery pigs with impaired intestinal health show reduced ability of nutrient digestion and thus reduced growth. A tremendous amount of research effort has been made to determine nutritional strategies to maintain or improve intestinal health and microbiota in nursery pigs. A large number of feed additives have been evaluated for their effectiveness on improving intestinal health and balancing intestinal microbiota in nursery pigs. Selected prebiotics, probiotics, postbiotics, and other bioactive compounds can be used in feeds to handle issues with intestinal health. Selection of these feed additives should aim modulating biomarkers indicating intestinal health. This review aims to define intestinal health and introduce examples of nutritional approaches to handle intestinal health in nursery pigs.

## INTRODUCTION

Since the first use of antibiotics for poultry in 1940’s [1–3] and for swine in 1950’s [4–6], they have been widely used for prophylaxis to handle issues with diseases as well as for growth promoting as antimicrobial growth promoters (AGP) in animal production [7–9]. However, due to emerging concerns over resistance to antibiotics for human, the use of AGP in animal feeds has been restricted or banned in several countries including Sweden (1986), Denmark (1995), EU (2006), Korea (2011), USA (2017), China (2020), and Vietnam (2020) [10–12].

**Figure 1:**
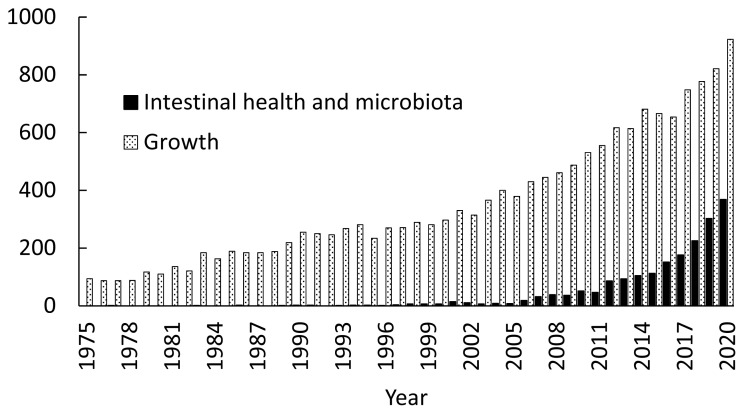
Number of peer-reviewed papers found in the PubMed. ■: using intestinal health and intestinal microbiota in pigs as keywords; ▨: using growth of pigs as keywords.

Increased occurrence of gut diseases with the removal of AGP in feeds, however, enlightens pig producers and animal scientists the importance of concepts of intestinal health [13,14], intestinal microbiota, and alternative feed additives. Importance of these topics is well shown by increased research activities. Simply searching the PubMed with keywords including intestinal health and microbiota in pigs provided over 1,900 peer-reviewed papers since 1960. However, 90% of all papers are published since 2010 indicating increased challenges with intestinal health and interests in intestinal microbiota of pigs especially during the last 10 years (Figure 1). Even after consideration of a general increase of research activities in swine nutrition and production, based on the number of peer-reviewed papers shown in the PubMed, research on intestinal health and microbiota is near 40% of general growth performance research in 2020, whereas it took less than 5% prior to 2007 or only less than 1% prior to 1997.

## INTESTINAL HEALTH

**Figure 2:**
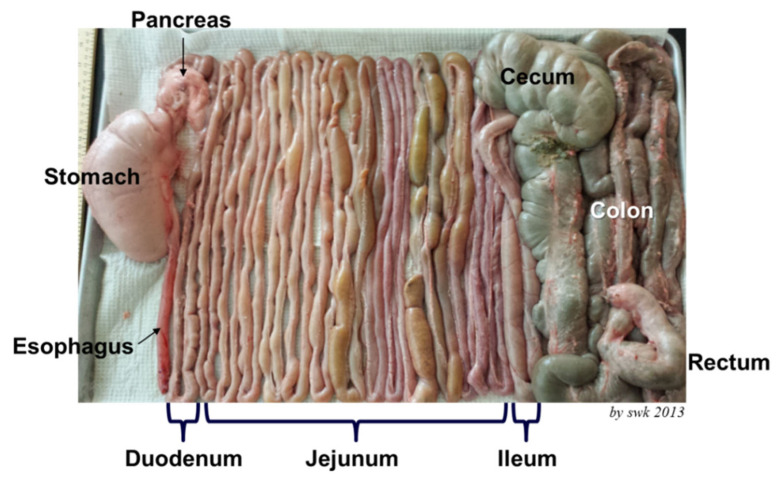
A photo of gastrointestinal tract of a pig at 5 weeks of age. The photo is taken by Sung Woo Kim in 2013 and also used in Eisemann and Kim [15].

The gastrointestinal tract is the largest and longest internal organ of pigs [15]. The intestine is separated into the small and large intestine. The small intestine is further divided to the duodenum, jejunum, and ileum. In pigs, the jejunum takes almost 80% of the small intestine (Figure 2) and where feeds are mostly digested to nutrients and then absorbed [13].

In the modern pig production, nursing piglets are weaned mostly at 3 to 4 weeks of age when they are too young considering that natural weaning occurs at around 8 to 10 weeks of age [16]. The intestine of early weaned pigs is premature with mucosal dysfunction [17,18]. This is the critical age for newly weaned pigs greatly affected by dietary factor. Conventional feeds given to newly weaned pigs include grains, legume seed meals, and animal byproducts. These feeds are essential sources of nutrients but also provide a notable amount of allergenic, antigenic, toxic, and pathogenic compounds to the intestine of newly weaned pigs [19–21]. Such stressors from the feeds often cause immune responses at the mucosal barrier of the jejunum followed by inflammatory responses and oxidative stress responses [22–26]. The jejunum is the major site of facing these challenges [15,18, 20,26] as well as for the feed digestion and nutrient absorption. Excessive inflammation and oxidative stress in the jejunum would cause destruction of villi and leaky gut resulting in reduced growth [24,27,28].

## EVALUATION OF INTESTINAL HEALTH

For the successful nutritional management of newly weaned pigs, therefore, prevention of unnecessary elevation of inflammation and oxidative stress in the jejunum would be the right direction. There can be several biological indicators to determine inflammatory status and oxidative stress status in the jejunum of pigs. Cytokines are small proteins secreted from cells for the communication among cells. Some cytokines (including tumor necrosis factor-α [TNF-α], interleukin-1β [IL-1β], IL-6, IL-8, and interferon-γ) functions as immunoregulatory molecules to activate immune cells causing inflammation and thus they are called pro-inflammatory cytokines [29], whereas cytokines (including IL-4, IL-10, and IL-13) reducing inflammatory responses are called anti-inflammatory cytokines [30]. Pro-inflammatory cytokines can be released from intestinal immune cells as well as mucosal epithelial cells [31,32] by increased production of free radicals causing oxidative stress [33]. Excessive free radicals can damage cellular components including cell membranes and intracellular enzymes. Malondialdehydes and carbonyls are products of oxidative damages of lipids and protein, respectively, which are well accepted biological markers for oxidative stress [34–36].

Increased intestinal inflammation and oxidative stress cause damages in the intestinal epithelium and the structure of villi [37–39]. Crypt cell proliferation follows to repair damages in the intestinal epithelium [40,41]. Crypt cell proliferation can be quantified using immunohistochemistry by staining Ki67 proteins in proliferating cells [27,42,43]. Collective outcomes of intestinal inflammation, oxidative stress, epithelial damages, and crypt cell proliferation affect nutrient digestibility and eventually growth of pigs [25,44,45].

A collective review of over 10 recent publications from our laboratory indicates that typical lean type nursery pigs with normal growth maintain 0.2 to 2.0 pg TNF-α and 0.2 to 0.8 μmol malondialdehydes per mg protein in the jejunal mucosa as selected examples. Increases of TNF-α and malondialdehydes showed negative correlation with growth of nursery pigs (Unpublished data). Nursery pigs with TNF-α greater than 0.6 pg and malondialdehydes greater than 0.5 μmol per mg protein in the jejunal mucosa showed reduced weight gain. This example shows that selected inflammatory and oxidative stress measurements of the jejunal mucosa could be effective biomarkers for the intestinal health and thus potential growth of nursery pigs. Then nutritional strategies to maintain or improve intestinal health of pigs upon weaning could target altering some of these biomarkers. Recent publications, evaluating the effectiveness of these nutritional strategies, extensively target to affect the status in the jejunum by reducing the excessive release of pro-inflammatory cytokines and oxidative stress products. This alternation would maintain healthy villus structure with effective barrier functions which will in turn influence potential growth of nursery pigs.

## JEJUNAL MUCOSA-ASSOCIATED MICROBIOTA

There has been an extended attention to understand the role of microbiota associated with the jejunal mucosa to maintain or improve the intestinal health of nursery pigs. The profile, so called relative abundance, of microbiota associated with intestinal mucosa is largely different from luminal microbiota associated with digesta in nursery pigs [46,47]. Their role or function to the host animal would be different. Luminal microbiota interacting with the digesta would affect nutrient digestion and secrete metabolites, whereas those associated to the intestinal mucosa are shown to crosstalk directly with intestinal immune cells [48–50] and prevent colonization of pathogenic bacteria [51]. There are increased efforts to provide research data characterizing jejunal mucosa-associated microbiota and dietary influences in pigs [27,52,53]. However, there is no clear understanding on the relationship between jejunal mucosa-associated microbiota and intestinal health in pigs and, therefore, statistical investigation of such relationships with recent data would provide critical tools to provide solutions for intestinal health of nursery pigs.

## NUTRITIONAL STRATEGIES TARGETING INTESTINAL HEALTH

Animal nutritionists have evaluated and tested numerous alternative bioactive compounds, extracts, microorganisms, and feed additives to replace the use of AGP. The major target of these alternatives has been affecting and improving intestinal health of pigs with some promising outcomes but still without complete answers to replace AGP [9,54,55]. Potential and partial success has been obtained from the use of prebiotics [56,57], probiotics or direct-fed microbials [53,58], postbiotics [59–61], phytobiotics [62–64], non-starch polysaccharide degrading enzymes (NSPases) [24, 65–67], functional amino acids [68–70], acidifiers or organic acids [71,72], and other bioactive compounds [38,73,74].

Prebiotics traditionally used in feeds include fructo-oligo-saccharides and manno-oligosaccharides, but also extend to xylo-oligosaccharides and milk oligosaccharides [57,75]. Recent research uses NSPases such as xylanase and mannanse as feed supplements in feeds to provide xylo-oligosaccharides and manno-oligosaccharides as sources of prebiotics in the intestine of pigs [25]. Probiotics has long been used in animal production. Effective probiotics are extensively lactogenic bacteria as they can reduce pH of the intestinal lumen and can potentially colonize or associate to the intestinal mucosa, together providing undesirable environment to pathogenic bacteria in the intestine. Recent research indicates that effective probiotic bacteria should associate to jejunal mucosa affecting immune cells in the small intestine [25,76]. Typical probiotics used in pig production include, but not limited to, *Bacillus licheniformis*, *Bacillus subtilis*, *Bifidobacterium thermophilum*, *Bifidobacterium bifidum*, *Lactobacillus acidophilus*, *Lactobacillus plantarum*, *Lactobacillus reuteri*, *Saccharomyces cerevisiae*, and *Enterococcus fascium*. Each strain of probiotics has a preferred environment based on availability of oxygen in the intestine of pigs. Effectiveness of probiotics with multiple strains should be compared with probiotics with a single strain. Postbiotics include bioactive compounds produced by probiotics during a fermentation process. This is a relatively new term but yeast culture [59,60] and yeast cell wall extracts [27,61] are examples of traditional postbiotics used in pig production.

Phytobiotics includes herbal extracts and essential oils that typically possess antioxidant, antibacterial, and antiviral properties [62–64,77]. Bioactive compounds in phytobiotics typically phenolic compounds with unique aroma [62]. Determination of an effective dose level would be an important item for the use of phytobiotics to prevent any potential negative impacts on feed intake. Feed enzymes degrading non-starch polysaccharides have been used in pig production mainly to provide additional nutrients by lessening cage effects of polysaccharides which entrap nutrients and feed particles in the small intestine of pigs [65–67]. However, recent research shows additional benefits of NSPases by converting NSP to potential prebiotics in the small intestine of pigs [24,25]. Some selected supplemental amino acids, such as arginine, glutamine, methionine, possess unique functions in the animal body. Methionine reduces oxidative stress in the intestinal epithelium [68], whereas glutamine prevents jejunal atrophy [78]. Arginine is also shown to be effective by its role in angiogenesis and vasodilation in the small intestine [79,80].

A combinational use of multiple alternatives providing potential synergic benefits, so called synbiotics, is leading a recent trend of pig nutrition research [25,81–83].

## CONCLUSION

Nursery pigs receive continuous challenges to the small intestine from dietary and environmental factors. Inflammation and oxidative stresses are initial factors impairing intestinal health and compromise potential growth of nursery pigs. Intestinal health status can be determined by quantification of selected inflammatory cytokines, oxidative stress products, and crypt cell proliferation in the jejunum. The profile of jejunal mucosa-associated microbiota are additional indicators of intestinal health warranting further research. Selected prebiotics, probiotics, postbiotics, and other bioactive compounds can be used in feeds to handle issues with intestinal health. Selection of these feed additives should aim modulating biomarkers indicating intestinal health and balancing intestinal microbiota.
